# Functional Activity of Antibodies Directed towards Flagellin Proteins of Non-Typhoidal *Salmonella*

**DOI:** 10.1371/journal.pone.0151875

**Published:** 2016-03-21

**Authors:** Girish Ramachandran, Sharon M. Tennant, Mary A. Boyd, Jin Y. Wang, Mohan E. Tulapurkar, Marcela F. Pasetti, Myron M. Levine, Raphael Simon

**Affiliations:** 1 Center for Vaccine Development, University of Maryland School of Medicine, Baltimore, Maryland, United States of America; 2 Department of Medicine, University of Maryland School of Medicine, Baltimore, Maryland, United States of America; 3 Department of Pediatrics, University of Maryland School of Medicine, Baltimore, Maryland, United States of America; 4 Division of Pulmonary and Critical Care, University of Maryland School of Medicine, Baltimore, Maryland, United States of America; University of Osnabrueck, GERMANY

## Abstract

Non-typhoidal *Salmonella* (NTS) serovars Typhimurium and Enteritidis are major causes of invasive bacterial infections in children under 5 years old in sub-Saharan Africa, with case fatality rates of ~20%. There are no licensed NTS vaccines for humans. Vaccines that induce antibodies against a *Salmonella* Typhi surface antigen, Vi polysaccharide, significantly protect humans against typhoid fever, establishing that immune responses to *Salmonella* surface antigens can be protective. Flagella proteins, abundant surface antigens in *Salmonella* serovars that cause human disease, are also powerful immunogens, but the functional capacity of elicited anti-flagellar antibodies and their role in facilitating bacterial clearance has been unclear. We examined the ability of anti-flagellar antibodies to mediate microbial killing by immune system components *in-vitro* and assessed their role in protecting mice against invasive *Salmonella* infection. Polyclonal (hyperimmune sera) and monoclonal antibodies raised against phase 1 flagellin proteins of *S*. Enteritidis and *S*. Typhimurium facilitated bacterial uptake and killing of the homologous serovar pathogen by phagocytes. Polyclonal anti-flagellar antibodies accompanied by complement also achieved direct bacterial killing. Serum bactericidal activity was restricted to *Salmonella* serovars expressing the same flagellin used as immunogen. Notably, individual anti-flagellin monoclonal antibodies with complement were not bactericidal, but this biological activity was restored when different monoclonal anti-flagellin antibodies were combined. Passive transfer immunization with a monoclonal IgG antibody specific for phase 1 flagellin from *S*. Typhimurium protected mice against lethal challenge with a representative African invasive *S*. Typhimurium strain. These findings have relevance for the use of flagellin proteins in NTS vaccines, and confirm the role of anti-flagellin antibodies as mediators of protective immunity.

## Introduction

Non-typhoidal *Salmonella* (NTS) serovars Enteritidis and Typhimurium are increasingly being recognized as major causes of invasive bacterial disease (sepsis, meningitis, etc.) in infants and toddlers in sub-Saharan Africa where ~ 20–25% of cases are fatal [1]. There are no licensed NTS vaccines, and the majority of African NTS isolates from invasive infections are resistant to multiple commonly used antibiotics [1, 2]. Development of an effective vaccine could serve as an important countermeasure and public health tool to reduce the burden of invasive NTS disease.

*Salmonella* are intracellular pathogens, and presumed to be shielded from the bactericidal effects of antibodies while they are sequestered within the host cell. They are, however, likely vulnerable to killing by antibodies when they are extracellular, including prior to invasion of host cells and following release after cell lysis [3]. Vaccines that have induced antibodies against *Salmonella* bacterial surface antigens have proven protective in animal models of invasive NTS infection (e.g., O polysaccharide glycoconjugates, outer membrane proteins [OMPs]) and in typhoid Vi capsule-based vaccine field trials [4–10].

*Salmonella* flagella are documented virulence factors and prominent surface structures that emanate from the cell surface as thin tubes that are up to 15 μm long, with *~*4–10 flagella per bacterium [11–13]. The central filament region that comprises the bulk of the flagellum structure is a multimer of up to 20,000 flagellin protein subunits [14]. *Salmonella* generally encode genes for either one (e.g., *S*. Enteritidis) or two (e.g., *S*. Typhimurium) flagellins, termed phase 1 (FliC) and phase 2 (FljB) flagellin, that are expressed discordantly. The distinct epitopes present within the different flagellin proteins are characteristic and conserved for each serovar and are used in conjunction with O polysaccharide epitopes for serotyping [15].

*Salmonella* flagellin proteins are highly immunogenic, inducing robust serum antibody responses in mice and man after wild-type infection or immunization with attenuated vaccine strains [9, 16, 17]. *Salmonella* flagellin subunits are also potent agonists of Toll-like Receptor 5 on intestinal epithelial cells, triggering inflammatory cytokine release and recruitment of immune effector cells that are targets for invasion and vehicles for systemic dissemination [18].

Although much is known about the mechanisms by which *Salmonella* flagellins induce T and B cell responses, little is known with respect to the functional properties of anti-flagellin antibodies [19–21]. Two important bactericidal mechanisms mediated by antibodies are activation of the complement system and enhancement of bacterial uptake by phagocytic cells. Microbial killing by the classical complement pathway is initiated through activation of C1 by IgG or IgM which leads to outer membrane pore formation by the C9 membrane attack complex (MAC). IgG antibodies also enhance uptake by phagocytes through Fc gamma receptors (FcGR), with subsequent killing by oxidative burst within the phagolysosome. Our goal here was to characterize and assess the capacity of antibodies specific for non-typhoidal *Salmonella* flagellin proteins to mediate anti-bacterial activity *in-vitro* by these pathways, and to provide functional protective activity *in-vivo*.

## Materials and Methods

### Bacterial strains, medium and reagents

The strains used in this study are described in Table 1. All strains were grown in Hi-Soy (HS) bacteriological media (5 g/L sodium chloride, 10 g/L soytone [Teknova, Hollister, CA], 5 g/L Hy-yest [Sigma Aldrich, St. Louis, MO]) at 37°C.

**Table 1:** Bacterial strains used in this study.

Serovar	Strain	Source/characteristics	References
***S*. Typhimurium**	D65	Clinical isolate, Mali	[22–24]
***S*. Enteritidis**	S15	Clinical isolate, Mali	[22–24]
***S*. Typhi**	Ty2	Wild-type	[25]
***S*. Paratyphi A**	ATCC 9150	Wild-type	American Type Culture Collection, Manassas, VA

### Preparation of purified flagellins used as immunogen

Flagellin preparations used to generate polyclonal anti-*S*. Enteritidis FliC sera for *in-vitro* studies were prepared from cell-associated flagella as previously described [9]. Flagellin preparations used to generate polyclonal anti-*S*. Typhimurium FliC sera, *S*. Typhimurium FliC monoclonal antibodies, and *S*. Eneritidis FliC monoclonal antibodies were generated from liquid growth culture supernatants of *S*. Enteritidis and *S*. Typhimurium as described elsewhere [26]. Flagellin preparations were confirmed for purity as described [9, 26] by SDS-PAGE with coomassie staining, and for endotoxin by the Limulus amebocyte lysate assay using the Endosafe PTS® system (Charles River, MA).

### Monoclonal antibodies

BALB/c mice were immunized with purified flagellins from *S*. Enteritidis or *S*. Typhimurium [26] and monoclonal antibodies were produced from hybridoma fusions (Antibody and immunoassay consultants, MD). Monoclonal antibodies were identified serologically as IgG1, and were purified from hybridoma cell culture supernatants with protein A resin (GE, New Jersey). The anti-flagellin specificity of each monoclonal antibody assessed by ELISA is detailed in Table 2 and shown in S1 Fig.

**Table 2:** Monoclonal IgG antibodies used in this study

Monoclonal antibody	Antibody specificity	IgG isotype	Reference
**CA6IE2**	*S*. Enteritidis phase 1 flagellin	IgG1	[26]
**JB11IG4**	*S*. Enteritidis phase 1 flagellin	IgG1	[26]
**AH12IE6**	*S*. Typhimurium phase 1 flagellin	IgG1	This study
**CB7IH2**	*Salmonella* flagellins	IgG1	[26]
**AE9IB4**	*Salmonella* flagellins	IgG1	[26]

### Mice

Female outbred CD-1 mice (8–10 week old) were purchased from Charles River Laboratories (Wilmington, MA). Animal protocols were approved by the University of Maryland School of Medicine Institutional Animal Care and Use Committee.

### Anti-flagellin sera

Sera were obtained from mice immunized IM as described [9], with two spaced doses of 2.5 μg of *S*. Enteritidis flagellin given 10 days apart, or three spaced doses of 2.5 μg of *S*. Typhimurium flagellin given 28 days apart. Serum samples were obtained 14–21 days after the final dose and stored at -20°C until use.

### Passive immunization and challenge

To assess the protective effect of passively transferred monoclonal anti-flagellin, naïve mice were injected intravenously (IV) through the tail vein with 100 μl of sterile PBS alone or containing 50 μg of monoclonal antibodies AH12IE6 or CA6IE2. Two to 3 hours later they were inoculated IP with 1 x 10^5^ CFU of *S*. Typhimurium D65. Challenged mice were monitored for 14 days, recording overall health, weight loss and mortality. Moribund mice exhibiting signs including lethargy, non-responsiveness and ≥ 20% weight loss were euthanized and scored as dead.

### Serum bactericidal antibody (SBA) activity assay

Assays were conducted as described using exogenous baby rabbit complement (BRC) (Pel-Freez Biologicals, AR) [27]. Mouse sera were heat-inactivated at 56°C for 20 min prior to use in the assay. Monoclonal antibodies and serum samples were prepared by two-fold serial dilution in normal saline in a 96-well plate. As target strains, *S*. Typhimurium D65, *S*. Enteritidis S15, *S*. Typhi Ty2 and *S*. Paratyphi A ATCC 9150 were used. Serum bactericidal activity titer was defined as the reciprocal of the highest serum dilution that produced > 50% killing in relation to the killing observed for the negative control containing only bacteria and complement (no serum or antibody).

### Opsonophagocytic activity (OPA) assay

HL-60 cells were maintained using RPMI supplemented with 10% [v/v] fetal bovine serum (FBS). Prior to use, cells were pre-washed once in HBSS and resuspended in opsonization buffer (OPB) (RPMI containing Mg^2+^ and Ca^2+^ [Life Technologies, Carlsbad], supplemented with 0.1% gelatin, and 10% FBS). Bacteria were prepared by performing a 1:1,000 dilution of an overnight culture in fresh HS media and incubating at 37°C, 160 RPM until the OD_600_ nm reached 0.3–0.45. Log phase cultures (~1 x 10^8^ CFU/ml) were then diluted in OPB to a concentration of ~1 x 10^5^ CFU/ml. Monoclonal antibodies were serially diluted two-fold in a 96-well plate. Approximately 700–1000 CFU of the bacteria were added to each well for opsonization at 37°C for 15 min in a 5% CO_2_ incubator, at which point 12.5% BRC (final concentration) and 4 x 10^5^ HL-60 cells were added to each well. The 96 well plate was incubated at 37°C (no CO_2_) with shaking agitation at 160 RPM for 45 mins. Bacteria were enumerated by performing viable counts. The negative control for each comparison lacked antibody. OPA titer was defined as the reciprocal of the highest serum dilution that produced > 50% killing in relation to the killing observed for the negative control (no serum or antibody added).

### Immunofluorescence and immunogold electron microscopy

Binding of polyclonal anti-*S*. Enteritidis FliC antibodies to the flagella of *S*. Enteritidis strain S15 was detected by immunofluorescence staining and immunogold labeling with gold nanoparticle conjugated secondary antibody specific for mouse IgG. Staining and imaging techniques were performed by the Core Imaging Facility at the School of Medicine, University of Maryland Baltimore.

### Statistical analysis

All statistical analyses were performed using GraphPad Prism version 6 (GraphPad Software, San Diego, CA). Statistical significance for OPA and SBA analyses were assessed using the two-tailed paired t-test (α = 0.05), comparing individual sera from mice immunized with flagellin proteins relative to the levels obtained with control sera. Survival analysis after passive immunization was assessed by the log-rank test. P-values of < 0.05 were considered statistically significant.

## Results

### Functional bactericidal activity of polyclonal anti-flagellin antibodies

**Fig 1:**
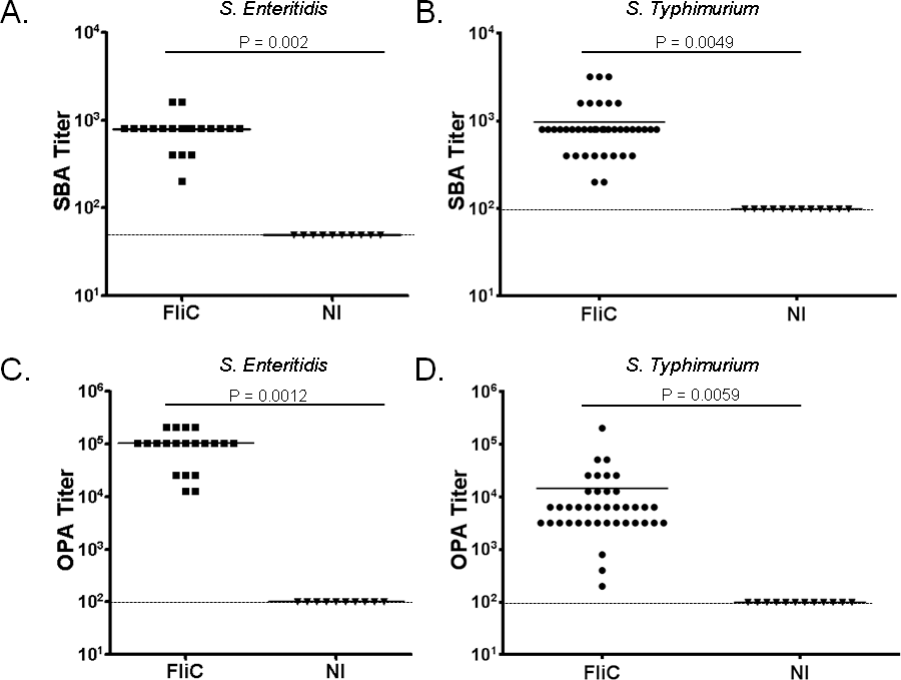
Endpoint SBA and OPA titers for sera from mice immunized with *S*. Enteritidis or *S*. Typhimurium phase 1 flagellin proteins. (A, B) SBA, and (C, D) OPA titers produced by individual mice immunized with *S*. Enteritidis phase 1 flagellin (A, C, closed squares), *S*. Typhimurium phase 1 flagellin (B, D, closed circles) or non-immune PBS controls (NI, closed triangles). * P value is indicated. Individual points are the average of triplicate wells from a single experiment. Solid bar = mean, dashed line = assay detection limit.

Sera from mice immunized intramuscularly (IM) with *S*. Enteritidis or *S*. Typhimurium FliC were assessed for complement-mediated serum bactericidal antibody (SBA) and opsonophagocytic antibody (OPA) activity. We observed significantly higher SBA and OPA titers in immune compared to control sera with end-point titers as high as 1:100,000 (Fig 1). The anti-flagellin sera demonstrated higher overall endpoint OPA titers than SBA titers.

### Anti-flagellin antibodies recognize surface associated flagella

**Fig 2:**
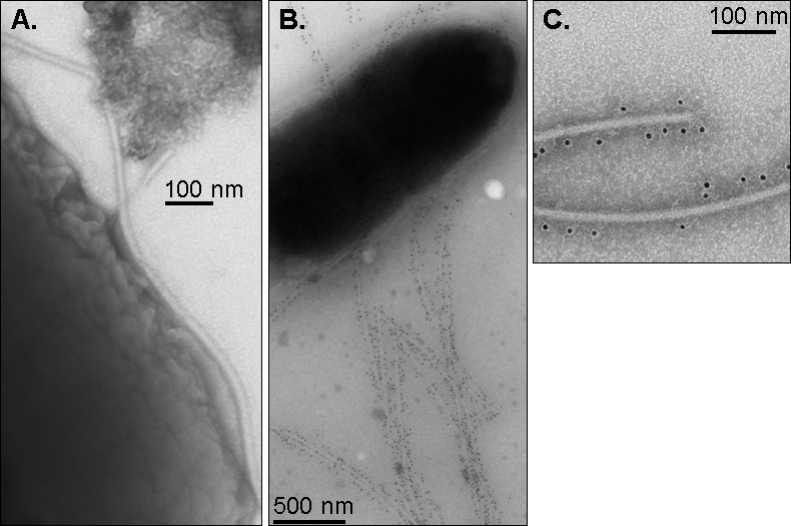
Immunogold labeling of *S*. Enteritidis strain S15 with polyclonal antisera against *S*. Enteritidis flagellin. Flagella labeled with (A) PBS control sera, (B,C) sera from mice immunized with *S*. Enteritidis phase 1 flagellin. Bars, 100 nm (A, C) and 500 nm (B).

The pattern of binding by polyclonal anti-*S*. Enteritidis FliC antibodies to whole flagella expressed by *S*. Enteritidis was investigated using immunogold electron microscopy, where it was revealed that anti-flagellin IgG antibodies were bound uniformly along the length of the flagella filament (Fig 2). The specificity of the antisera for flagellin was confirmed by lack of reactivity with an *S*. Enteritidis Δ*fliC* strain in a western blot (S2 Fig).

### Assessment of SBA activity for heterologous *Salmonella* serovars with polyclonal sera raised against *S*. Enteritidis FliC

**Fig 3:**
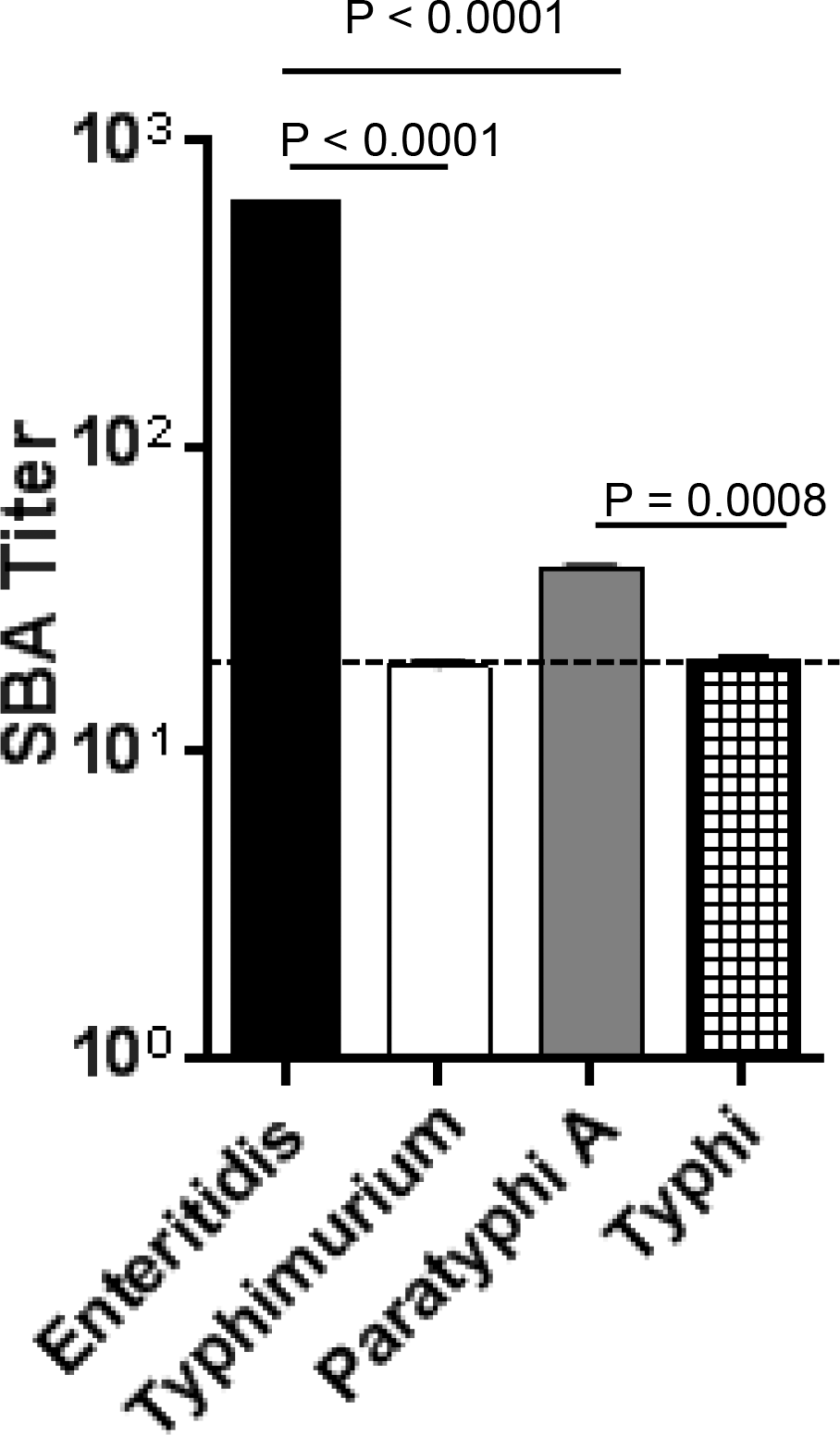
Comparison of SBA titers of *S*. Enteritidis anti-FliC polyclonal antibodies against heterologous *Salmonella* serovars. SBA titer produced by pooled sera (n = 10) from mice immunized with *S*. Enteritidis phase 1 flagellin against the target strains *S*. Enteritidis S15, *S*. Typhimurium D65, *S*. Paratyphi A ATCC9150, and *S*. Typhi Ty2. Dashed line = detection limit. Data shown is the average and standard deviation of triplicate wells, and representative of two independent experiments. P values were assessed by paired t-test.

*Salmonella* isolates are classified according to the Kaufmann-White serotyping scheme that is based on the unique epitopes in their O polysaccharide (LPS) and H (flagellin) antigens [15]. Since sera from mice immunized with purified FliC from *S*. Enteritidis (O epitopes:1,9,12; H epitopes:g,m) caused killing of the homologous serovar by complement, we wanted to determine whether complement-mediated bactericidal activity could also be achieved against *Salmonella* serovars that express heterologous flagellin types. For this, SBA assays were performed incubating pooled anti-H:g,m sera, raised against purified *S*. Enteritidis FliC, with *Salmonella* serovars Paratyphi A (O epitopes:1,2,12; H epitope:a) and Typhimurium (O epitopes:1,4,[5],12; H epitopes:i:1,2), which express different O and H antigens, or Typhi (O epitopes:9,12; H epitope:d), which shares the same immunodominant O polysaccharide determinant (epitope 9) but has a different flagellin type than *S*. Enteritidis. We found no detectable SBA activity against *S*. Typhimurium or *S*. Typhi using anti-H:g,m sera, and only limited killing of *S*. Paratyphi A (Fig 3). Interestingly, despite the paucity of functional cross-reactivity by anti-*S*. Enteritidis flagellin antibodies, measurable antibody binding was detectable by ELISA for the phase 1 flagellin proteins of the heterologous serovars with geometric mean titers (GMT) approaching 20% of the *S*. Enteritidis FliC GMT (S3 Fig).

### Functional antibacterial activity of anti-flagellin monoclonal antibodies *in-vitro*

**Fig 4:**
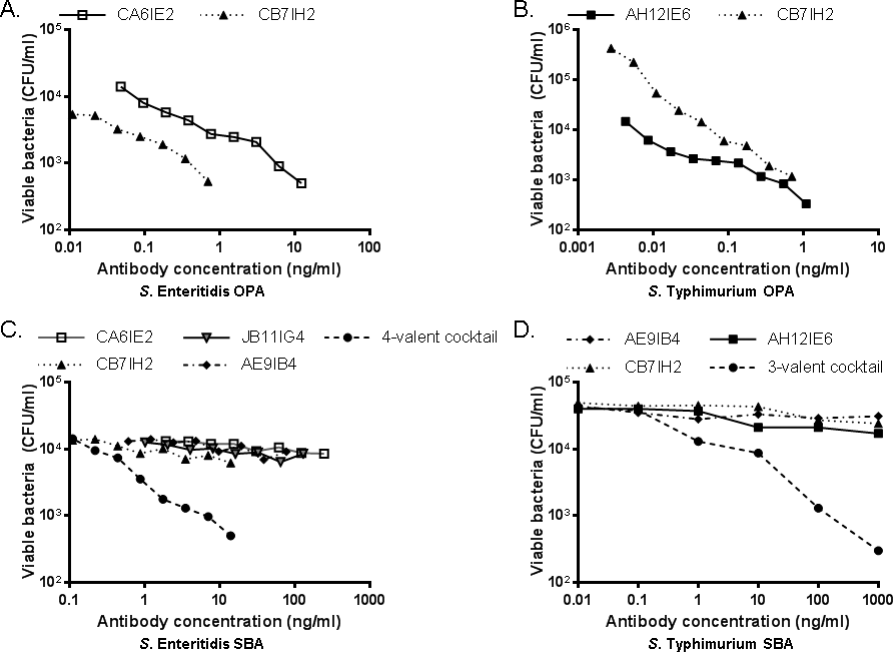
OPA and SBA activity for monoclonal antibodies specific for the phase 1 flagellin proteins of *S*. Enteritidis and *S*. Typhimurium. OPA was assessed for (A) *S*. Enteritidis S15 and (B) *S*. Typhimurium D65 with phase 1 serovar specific (CA6IE2 [*S*. Enteritidis]; AH12IE6 [*S*. Typhimurium]) and broadly-reactive (CB7IH2) anti-flagellin antibodies. SBA was assessed for (C) *S*. Enteritidis S15 and (D) *S*. Typhimurium D65 with phase 1 serovar specific (CA6IE2, JB11IG4 [*S*. Enteritidis]; AH12IE6 [*S*. Typhimurium]),) and broadly-reactive (CB7IH2, AE9IB4) anti-flagellin antibodies, tested individually or mixed in equal amounts. The total protein concentration of the individual or combined antibodies is indicated. Each point is the average of triplicate wells from a single experiment.

To assess the functional activity of anti-flagellin antibodies at the level of single epitope specificity and in the absence of other serum components, different anti-flagellin monoclonal antibodies were tested in SBA and OPA assays. For these analyses, increasing concentrations of various IgG1 antibodies documented as type-specific or broadly reactive with flagellins from different *Salmonella* serovars (Table 2, S1 Fig) were examined for their capacity to mediate SBA and OPA against various *Salmonella* strains. Monoclonal antibodies specific for phase 1 flagellins from *S*. Enteritidis (CA6IE2) or *S*. Typhimurium (AH12IE6) mediated robust OPA activity when tested individually against strains expressing the homologous flagellin type. A broadly cross-reactive monoclonal antibody, CB7IH2, also mediated OPA activity against both *S*. Enteritidis and *S*. Typhimurium (Fig 4A and 4B). Unexpectedly, whereas polyclonal antisera manifested measurable SBA activity, individual monoclonal antibodies did not produce detectable killing of *S*. Enteritidis or *S*. Typhimurium by complement, including at concentrations that were 100-fold greater than those found to cause robust enhancement of OPA (Fig 4C and 4D). Strikingly, SBA activity was robust when multiple monoclonal antibodies were combined in equal proportions.

### Monoclonal anti-*Salmonella* flagellin IgG maintains functional protective capacity *in-vivo*

**Fig 5:**
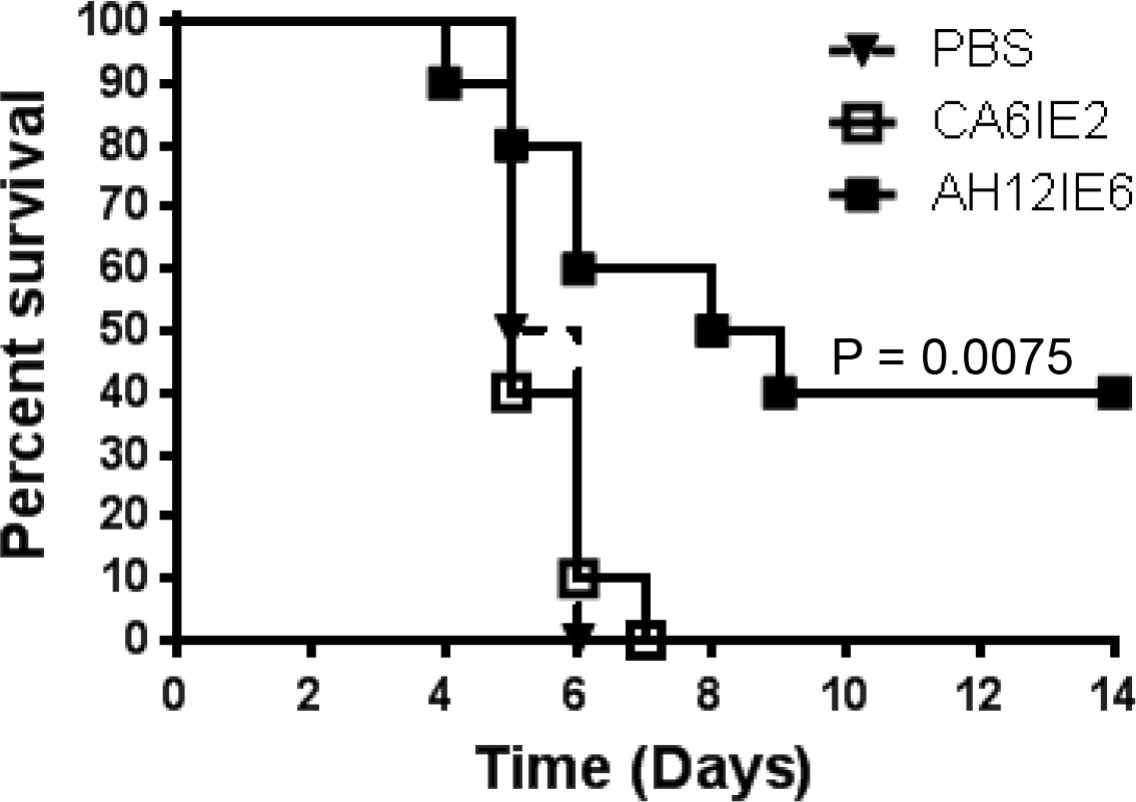
Passive immunization with monoclonal FliC antibodies and protection against lethal challenge. CD-1 mice (n = 10 /group) were intravenously administered PBS or 50 μg of either a monoclonal IgG specific for *S*. Enteritidis FliC (CA6IE2) or a monoclonal IgG specific for *S*. Typhimurium FliC (AH12IE6). Two to 3 hours later, they were infected intraperitoneally with 1 x 10^5^ CFU of *S*. Typhimurium D65, and monitored for 14 days recording mortality and overall health. P value by log-rank survival analysis versus control mice administered CA6IE2.

To determine whether anti-*Salmonella* flagellin IgG can effect functional activity *in-vivo*, mice were passively immunized with monoclonal antibody AH12IE6, that is mono-specific for *S*. Typhimurium phase 1 flagellin. Control mice were given either PBS or a monoclonal antibody specific for *S*. Enteritidis phase 1 flagellin (CA6IE2). Two to 3 hours later, mice were then infected intraperitoneally with 1 x 10^5^ CFU of *S*. Typhimurium D65. Mortality was recorded until 14 days after which point no further deaths occurred. Mice that were passively transferred *S*. Typhimurium FliC-specific monoclonal antibody were significantly protected (P < 0.05) compared to mice administered PBS or *S*. Enteritidis FliC-specific monoclonal antibody (Fig 5).

## Discussion

Although anti-flagella antibodies are major constituents of the immune response to *Salmonella* [16, 20], their protective efficacy and functional immunological activity have heretofore been poorly defined. We previously reported that *S*. Enteritidis phase 1 flagellin is a protective antigen and effective carrier protein for chemically linked core and O polysaccharide (COPS) from the same serovar [9]. Since flagellin proteins are abundant surface expressed proteins on *Salmonella*, we postulated that anti-flagellin antibodies could mediate functional anti-bacterial activity and possibly enhance the protective efficacy of the vaccine [9, 17, 28, 29].

We found here that polyclonal anti-flagellin sera and monoclonal anti-flagellin antibodies facilitated OPA killing of *Salmonella*. Antibodies opsonize microbial pathogens and enhance phagocytic activity through binding to the FcGR on activated macrophages and phagocytic cells. Immunogold electron microscopy revealed that anti-flagellin IgG antibodies bound along the length of the *Salmonella* flagellar filament, producing large protracted surfaces that serve as ligands for FcGR’s. Killing by oxidative burst within phagocytes plays an important functional role in host defense against invasive bacteria, contributing to resolution of disseminated *Salmonella* infections [30]. Indeed, OPA may be a common effector mechanism of anti-flagella antibodies, as prior studies have documented enhanced uptake of *Pseudomonas aeruginosa* by polyclonal rabbit sera raised against the homologous flagellin serotype [31].

For functional bactericidal activity by complement to occur, adjacent antibodies must activate the classical complement pathway at a point proximal to the bacterial surface in order for the MAC to form within the cell membrane. Flagella demonstrate remarkable structural flexibility, and the required proximity between antigen-antibody complexes and the cell surface necessary for MAC formation could occur when flagella lay flat or are wrapped around the cell. We found that polyclonal anti-flagellin sera mediated SBA activity. To our knowledge, this is the first report of anti-flagellar antibodies mediating direct killing in the presence of complement. SBA activity was found to be largely restricted to the homologous flagellin immunogen. The serovar specific amino acid sequences in the variable domain of flagellin form the outer filament surface, and are presumed to be the main target for anti-bacterial antibodies [14, 32]. The lack of cross-serovar SBA killing is thus consistent with the notion that the conserved residues of flagellin are largely buried within flagella and are poorly accessible to antibodies [14]. This is supported by a prior report that found monoclonal antibodies specific for conserved flagellin epitopes were able to bind flagellin proteins in ELISA and western blot assays, but failed to agglutinate bacteria indicating shielding of these epitopes when they were incorporated into flagella [33]. We similarly found that polyclonal anti-sera against *S*. Enteritidis phase 1 flagellin (H:g,m) demonstrated measurable ELISA titers for heterologous flagellins purified from the same serovars that had no detectable reduction in viable CFU by SBA analysis. We did, however, observe that this sera caused a partial reduction in viable *S*. Paratyphi A CFU (H:a). As we have found that monoclonal antibodies specific for H:g,m exhibit a low level of cross-reactivity with purified H:a (S1 Fig), it is possible that the low level of anti-*S*. Paratyphi A activity could be accounted for by a weakly cross-reactive epitope in the type specific region of these two flagellins.

Unexpectedly, we found that individual monoclonal anti-flagellin antibodies failed to induce detectable SBA activity. Flagella filaments are hollow helical polymers comprised of uniform stacked arrays of flagellin subunits whereby 11 flagellin protofilament units form each full helical turn [14, 34]. The lack of SBA activity for individual monoclonal antibodies could possibly be accounted for by failure to achieve the multivalency required for complement activation, as antibodies bound to adjacent protofilament subunits may not achieve the necessary density, or spatial orientation for effective complement activation. Strongly supporting this hypothesis is the observation that SBA activity was restored when anti-flagellin monoclonal antibodies were administered as a multivalent mixture. The polypeptide surface that is accessible to antibodies is likely formed by multiple contiguous and overlapping epitopes that each constitute a unique antibody binding site. Polyclonal anti-flagellin antibodies could thus be tightly clustered at adjacent epitopes on the same monomer subunit, providing sufficient proximity for initiation and propagation of the complement cascade.

*Salmonella* are facultative intracellular pathogens, and maintain differential expression of virulence genes based on the physiological environment within the host. In *S*. Typhimurium, flagella expression is downregulated while they reside inside animal cells [35]. Nevertheless it is possible that cell-associated flagella may be present and accessible to antibodies during periods when the bacteria are extracellular. This includes the early stages of *Salmonella* infection prior to invasion of host cells, or upon release by pyroptotic host-cell lysis following dissemination to secondary sites of infection [36]. Protection by NTS vaccines after peroral *Salmonella* infection in highly susceptible inbred mouse strains requires the dual action of cellular and humoral immunity [4]. Active immunization studies with *S*. Typhimurium flagellin proteins in this model have documented survival against fatal infection and reduction in bacterial burden [20, 21]. We have found that live attenuated *S*. Enteritidis vaccines that elicited high anti-FliC serum IgG titers provided better protection against lethal challenge in this model than those that induced only moderate anti-FliC antibody titers [17]. Protection against fatal NTS infection by antibodies alone can be assessed by parenteral inoculation of highly virulent NTS in naturally *Salmonella* “resistant” mice [4]. We have documented previously that immunization with *S*. Enteritidis flagellin or passive transfer of post-vaccination sera from mice immunized with *S*. Enteritidis COPS:flagellin conjugates protected resistant outbred mice against fatal intraperitoneal *S*. Enteritidis infection [4, 9, 28]. We elected to assess whether a monoclonal antibody against FliC would protect based on the prior finding that *S*. Typhimurium phase 1 flagella are more important for virulence in mice than phase 2 flagella, as well as the observation that phase 2 flagellin deficient *S*. Typhimurium (1,4,[5],12:i:-) comprise a measurable proportion of invasive clinical NTS isolates in sub-Saharan Africa [24, 37]. We found here that passive transfer of a monoclonal IgG specific for *S*. Typhimurium phase 1 flagellin (AH12IE6) was protective in this challenge model against *S*. Typhimurium infection. Protection was verified as type specific, as monoclonal IgG CA6IE2 that is specific for *S*. Enteritidis phase 1 flagellin did not protect. As AH12IE6 manifested robust OPA activity, but no SBA killing by itself, it is presumed that the protective function *in-vivo* was by OPA.

Our findings here, that anti-*Salmonella* flagellin antibodies have functional anti-bacterial activity, establish a role for humoral immunity against flagella in protection against NTS infection. These findings further support the rationale for inclusion of flagellin in candidate NTS vaccine formulations. Clinical studies with flagellin based vaccines will be required however to confirm whether flagellin proteins are viable protective targets against disseminated *Salmonella* infections in humans.

## Supporting Information

S1 Fig Reactivity of monoclonal antibodies used in this study against phase 1 FliC proteins from different *Salmonella* serovars. ELISA reactivity of anti-flagellin antibodies used in this study with FliC proteins from different *Salmonella* serovars.
(TIF)

S2 Fig SDS-PAGE and Western blot with polyclonal anti-*S*. Enteritidis flagellin sera and R11. Bacterial lysates of *S*. Enteritidis R11(1), R11 Δ*fliC* (2), and 12 μg purified *S*. Enteritidis FliC (3) were analyzed by SDS-PAGE and Western blot using (A) monoclonal antibody CB7IH2 and (B) *S*. Enteritidis FliC polyclonal antisera. M = Molecular weight marker.
(TIF)

S3 Fig Comparison of IgG ELISA titers in anti-*S*. Enteritidis FliC polyclonal sera with heterologous *Salmonella* serovar flagellins. IgG ELISA titers (geometric mean, left axis) produced by individual sera from mice immunized with *S*. Enteritidis flagellin (n = 10) or PBS (n = 10), against phase 1 flagellins purified from *S*. Enteritidis S15, *S*. Typhimurium D65, *S*. Paratyphi A ATCC9150, and *S*. Typhi Ty2. The percent titer relative to the geometric mean anti-*S*. Enteritidis flagellin titer (cross-reactive titer) is given on the right axis. Results are expressed as the geometric mean +/- standard error. *, P < 0.05 by paired t-test versus *S*. Enteritidis.
(TIF)

S1 File Supporting information Methods and References. (DOCX)
