# Supplementary figures and images for: Functional Activity of Antibodies Directed towards Flagellin Proteins of Non-Typhoidal Salmonella
Source: PLoS One. 2016 Mar 21;11(3):e0151875. doi: 10.1371/journal.pone.0151875 (PMC4801366; doi:10.1371/journal.pone.0151875)

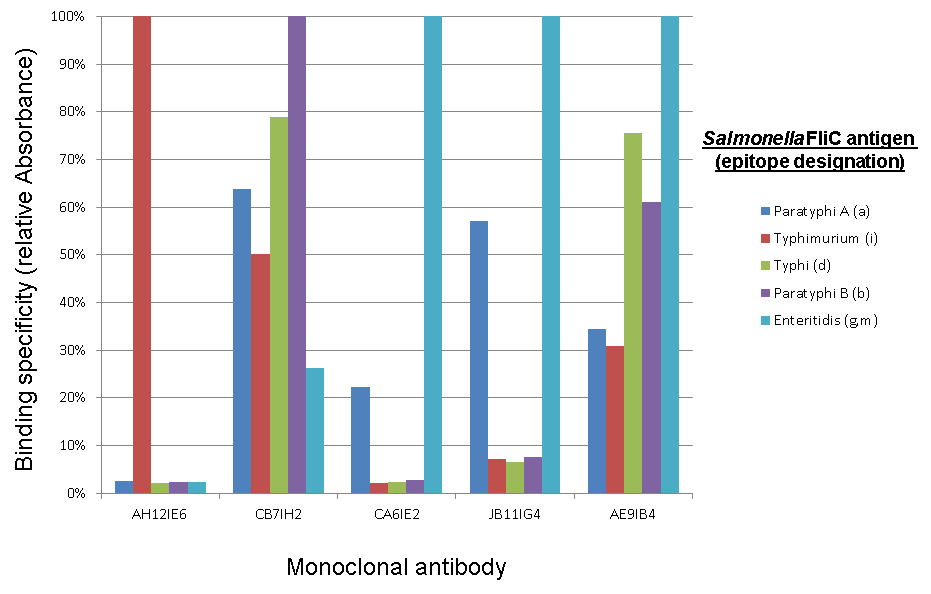

Supplement: S1 Fig — ELISA reactivity of anti-flagellin antibodies used in this study with FliC proteins from different Salmonella serovars. (TIF) [file pone.0151875.s001.TIF]

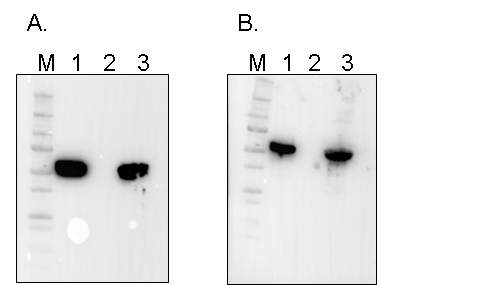

Supplement: S2 Fig — Bacterial lysates of S. Enteritidis R11(1), R11 ΔfliC (2), and 12 μg purified S. Enteritidis FliC (3) were analyzed by SDS-PAGE and Western blot using (A) monoclonal antibody CB7IH2 and (B) S. Enteritidis FliC polyclonal antisera. M = Molecular weight marker. (TIF) [file pone.0151875.s002.TIF]

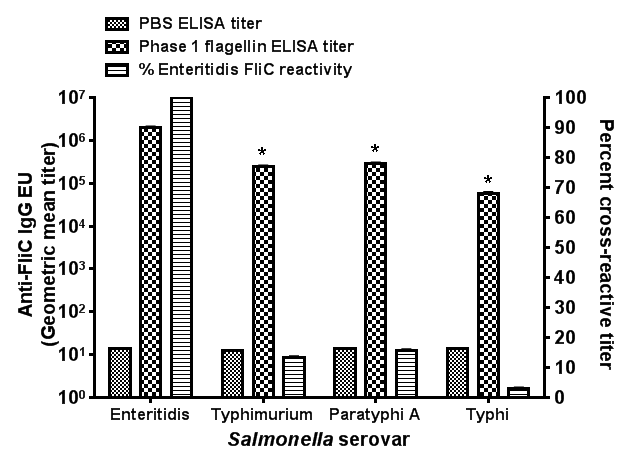

Supplement: S3 Fig — IgG ELISA titers (geometric mean, left axis) produced by individual sera from mice immunized with S. Enteritidis flagellin (n = 10) or PBS (n = 10), against phase 1 flagellins purified from S. Enteritidis S15, S. Typhimurium D65, S. Paratyphi A ATCC9150, and S. Typhi Ty2. The percent titer relative to the geometric mean anti-S. Enteritidis flagellin titer (cross-reactive titer) is given on the right axis. Results are expressed as the geometric mean +/- standard error. *, P < 0.05 by paired t-test versus S. Enteritidis. (TIF) [file pone.0151875.s003.TIF]
