# Supplementary material for: Functional Activity of Antibodies Directed towards Flagellin Proteins of Non-Typhoidal Salmonella
Source: PLoS One. 2016 Mar 21;11(3):e0151875. doi: 10.1371/journal.pone.0151875 (PMC4801366; doi:10.1371/journal.pone.0151875)
Supplement: S1 File — (DOCX) [file pone.0151875.s004.docx]

**Methods**

**Preparation of purified flagellins**

Flagellin preparations used as antigens for ELISA were prepared from cell-associated flagella as previously described [1].

**ELISA**

Analyses of binding to various flagellin proteins by monoclonal anti-flagellin antibodies and polyclonal sera were measured by ELISA, with end-point titers reported as ELISA units (EU)/ml, as previously described [1].

**Western immunoblot analysis of flagellin expression in bacterial lysates**

Overnight bacterial cultures were normalized to an OD_600_ of 0.2, from which a 1 mL aliquot was centrifuged at 4°C for 10 min at 13,200 rpm. Pellets or purified flagellin were resuspended in 100 µL of SDS-PAGE loading buffer (Biorad, CA), vortexed vigorously and heated at 100°C for 10 min from which 20 µL was loaded per well. Samples were separated on 4–20% gradient SDS-PAGE (Biorad, CA) and transferred to a PVDF membrane. The blots were blocked overnight with PBST and powdered milk, and then incubated for 1 h in a 1∶5000 dilution of FliC monoclonal CB7IH2 antibody or 1:1000 dilution of *S*. Enteritidis FliC polyclonal antisera. Bound antibodies were then detected with goat-anti-mouse secondary antibody (Cell Signaling, MA) and visualized with ECL reagent (Amersham/GE, NJ).

**References**

1. Simon R, Tennant SM, Wang JY, Schmidlein PJ, Lees A, Ernst RK, et al. *Salmonella enterica* serovar Enteritidis core O polysaccharide conjugated to H:g,m flagellin as a candidate vaccine for protection against invasive infection with *S*. Enteritidis. Infect Immun. 2011;79(10):4240-9. doi: 10.1128/IAI.05484-11. PubMed PMID: 21807909; PubMed Central PMCID: PMC3187246.
